# Prediction of dyslipidemia using gene mutations, family history of diseases and anthropometric indicators in children and adolescents: The CASPIAN-III study

**DOI:** 10.1016/j.csbj.2018.02.009

**Published:** 2018-03-02

**Authors:** Hamid R. Marateb, Mohammad Reza Mohebian, Shaghayegh Haghjooy Javanmard, Amir Ali Tavallaei, Mohammad Hasan Tajadini, Motahar Heidari-Beni, Miguel Angel Mañanas, Mohammad Esmaeil Motlagh, Ramin Heshmat, Marjan Mansourian, Roya Kelishadi

**Affiliations:** a Department of Biomedical Engineering, Facultyof Engineering, University of Isfahan, Isfahan, Iran; b Department of Automatic Control, Biomedical Engineering Research Center, Universitat Politècnica de Catalunya, BarcelonaTech (UPC), Barcelona, Spain; c Applied physiology researchcenter, Isfahan cardiovascular research institute, Isfahan University of Medical Sciences, Isfahan, Iran; d Department of Clinical Biochemistry, Tarbiat Modares University, Tehran, Iran; e Nutrition Department, Child Growth and Development Research Center, Research Institute for Primordial Prevention of Non-Communicable Disease,Isfahan University of Medical Sciences, Isfahan, Iran; f Biomedical Research Networking Center in Bioengineering, Biomaterialsand Nanomedicine (CIBER-BBN), Barcelona, Spain; g Department of Pediatrics, Ahvaz Jundishapur University of MedicalSciences, Ahvaz, Iran; h Department of Epidemiology, Chronic Diseases Research Center, Endocrinology and MetabolismPopulation Sciences Institute, Tehran University of Medical Sciences, Tehran, Iran; i Biostatistics and Epidemiology Department, Faculty of Health, Isfahan University of Medical Sciences, Isfahan, Iran; j Pediatrics Department, Child Growth and Development Research Center, Research Institute for Primordial Prevention of Non-Communicable Disease, Isfahan University of Medical Sciences, Isfahan, Iran

**Keywords:** Computer-assisted diagnosis, Deep learning, Dyslipidemia, Genomics, Health promotion, Machine learning

## Abstract

Dyslipidemia, the disorder of lipoprotein metabolism resulting in high lipid profile, is an important modifiable risk factor for coronary heart diseases. It is associated with more than four million worldwide deaths per year. Half of the children with dyslipidemia have hyperlipidemia during adulthood, and its prediction and screening are thus critical. We designed a new dyslipidemia diagnosis system. The sample size of 725 subjects (age 14.66 ± 2.61 years; 48% male; dyslipidemia prevalence of 42%) was selected by multistage random cluster sampling in Iran. Single nucleotide polymorphisms (rs1801177, rs708272, rs320, rs328, rs2066718, rs2230808, rs5880, rs5128, rs2893157, rs662799, and Apolipoprotein-E2/E3/E4), and anthropometric, life-style attributes, and family history of diseases were analyzed. A framework for classifying mixed-type data in imbalanced datasets was proposed. It included internal feature mapping and selection, re-sampling, optimized group method of data handling using convex and stochastic optimizations, a new cost function for imbalanced data and an internal validation. Its performance was assessed using hold-out and 4-foldcross-validation. Four other classifiers namely as supported vector machines, decision tree, and multilayer perceptron neural network and multiple logistic regression were also used. The average sensitivity, specificity, precision and accuracy of the proposed system were 93%, 94%, 94% and 92%, respectively in cross validation. It significantly outperformed the other classifiers and also showed excellent agreement and high correlation with the gold standard. A non-invasive economical version of the algorithm was also implemented suitable for low- and middle-income countries. It is thus a promising new tool for the prediction of dyslipidemia.

## Highlights

• Dyslipidemia is an important modifiable risk factor for coronary heart diseases.
• Its prediction and screening are critical in children and adolescents.
• We designed a new dyslipidemia diagnosis system as a new classification framework.
• Gene mutations, genetic, and anthropometric attributes were analyzed.
• The proposed framework showed excellent agreement with the gold standard.

## 1 Introduction

Strengthening the capacity of the entire countries, for early warning, and health risk reduction is one of the targets of the Sustainable Development Goal (SDG) #3. Non-communicable diseases (NCDs) have adverse human, social and economic consequences in all societies. Also, the first global NCD Action Plan is “A 25% relative reduction in the overall mortality from cardiovascular diseases, cancer, diabetes, or chronic respiratory diseases” [1]. Coronary heart diseases (CHDs), are the number 1 source of death and disability in countries including Iran [1,2]. Dyslipidemia, the disorder of lipoprotein metabolism resulting in high lipid profile, is a major risk factor of CHD [3]. It is related to more than four million deaths per year [4]. The accurate and reliable prediction of dyslipidemia is thus important in targeting SDG #3 and NCD Action Plan #1.

Metabolic risk factors including dyslipidemia are the most important determinants of emerging NCDs worldwide [5,6]. Dyslipidemia is, in fact, an important modifiable risk factor for CHD [7]. Although significant adverse health outcomes in childhood are not associated with dyslipidemia, it was shown in the literature that there is a link between childhood dyslipidemia and occurrence of atherosclerosis and its follow-up in adulthood [8,9]. Not only 40–55% of children with dyslipidemia will have hyperlipidemia during adulthood [10], but also subclinical atherosclerotic abnormalities, resulting in cardiovascular disease (CVD) events, occur in childhood [11]. Prediction and screening dyslipidemia, an important CVD risk factor, in children and adolescents is thus critical [12].

Some studies were performed in the literature to assess the genetic risk for dyslipidemia [13,14]. In such studies, statistically significant dyslipidemia predictors were identified, and no actual prediction (or classification) was performed. CAD (Computer-aided diagnosis), on the other hand, could use risk factors and predict if a subject is at high risk or not. CAD, which is using data mining to interpret medical information, could improve the diagnosis accuracy [15]. CAD is in fact used as a second opinion by the physicians to make the final diagnosis or prognosis decision [[16], [17], [18]].

Two methods were proposed in the literature to predict dyslipidemia in adults [19,20]. Wang et al. [19] analyzed 8914 subjects aged 35–78 years (with the prevalence of dyslipidemia about 46%). The predictors' age, gender, occupation, education, marital status, physical activity, individual income, waist circumference, smoking, family history of dyslipidemia, and diet were used to predict dyslipidemia (High TC, or TG or low HDL-C [21]). Artificial neural network (ANN) and Multiple Logistic Regression (MLR) models were used and the sensitivity, specificity, and precision of 90%, 77%, and 76% were obtained in the hold-out (75%) internal validation.

Costanza and Paccaud [20], analyzed 2549 subjects aged 35–64 years (the prevalence of dyslipidemia about 43%). The predictors waist-to-hip circumference ratio (WHR), body mass index (BMI), gender, age, current cigarette Smoking, and high blood pressure were used and dyslipidemia (total serum cholesterol to high-density lipoprotein cholesterol (TC/HDL-C) ratio ≥5.0) was predicted using different data mining methods, namely as the linear and logistic regressions, regression and classification trees. The sensitivity, specificity, and precision of 70%, 77%, and 69% were obtained in the hold-out external validation.

Although the prediction methods proposed in [19,20], are simple and effective and thus worthwhile for the identification of high risk people for having dyslipidemia based on the demographic, dietary and life-style, and anthropometric data, an optimal prediction is still required. Genome-based prediction of diseases has been recently focused in bioinformatics [22]. Identifying genetic mutations could assist in choosing optimal patient treatment. In fact, a lot of methods exist to reveal such mutations, including next-generation sequencing and future commercially available kits [23]. Moreover, in reliable clinical systems, critical criteria regarding statistical errors, precision, and DOR (Diagnosis Odds Ratio) must be met [24]. Moreover, considering ethnic differences in life-style, environmental factors and genetic background, examining gene polymorphisms associated with dyslipidemia in each ethnic group is important [13].

The purpose of our work is thus to design an *accurate* and *reliable* system for the prediction of dyslipidemia using gene mutations, family history of diseases and anthropometric indicators in a nationally-representative sample of the pediatric population in the Middle East and North Africa (MENA). To the best of our knowledge, this is the first study of its kind for genome-based dyslipidemia prediction using data mining.

## 2 Material and methods

### 2.1 Study population

The third study of a school-based surveillance system known as the childhood and adolescence surveillance and prevention of Adult Noncommunicable disease (CASPIAN) was conducted in Iran as the national survey of school students with high-risk behaviors (2009–2010) [25]. The description of the CASPIAN-III study was provided elsewhere in details [25]. Here, it is briefly described.

Among the youngsters, long-term changes in disease patterns are following rapid modifications in lifestyle, nutrition, and physical activity. Iranian youths are experiencing such lifestyle changes, making them prone to risk factors of chronic diseases such as NCDs. Surveilling such factors is important for long-term national planning based on monitoring NCD-related risk factors from childhood to adulthood. A school-based surveillance system entitled as CASPIAN Study was implemented in IRAN from 2003–2004. The surveys have been repeated every 2 years, with blood sampling for biochemical factors every 4 years.

This study was performed among 5570 students, sampled from 27 provinces of Iran. The entire students and their parents gave informed consent to the experimental procedure. It was approved by Isfahan University of Medical Sciences Panel on Medical Human Subjects and conformed to the Declaration of Helsinki.

According to the US National Institutes of Health Heart, Lung, and Blood Institute (NHLBI) guideline, which is one the acceptable criteria, dyslipidemia was defined for children and Adolescents (age ≤ 19 years) as having at least one of the following: TC (total cholesterol) ≥5.17 mmol/L (≥200 mg/dL), LDL-C (low-density lipoprotein cholesterol) ≥3.36 mmol/L (≥130 mg/dL), HDL-C (high-density lipoprotein cholesterol) levels <1.04 mmol/L (<40 mg/dL), TG (triglyceride) ≥1.13 mmol/L (≥100 mg/dL) when age is between zero and nine years and TG ≥1.47 mmol/L (≥130 mg/dL) when age is between 10 and 19 years, and finally non-HDL-C (subtracting HDL-C from TC) ≥3.75 mmol/L (≥145 mg/dL) [7,26].

We randomly selected 725 frozen whole blood samples for genome analysis from children and adolescents (48% male, 42% prevalence of dyslipidemia) taken from CASPIAN-III study. Such a sample size was estimated based on the sample-size estimation method proposed by Hajian-Tilaki [27]. Total required sample size (*N*) could be estimated based on the target sensitivity (Se_e_) and Specificity (Sp_e_) using Eq.(1):

(1) $$N=\max\left(\frac{z_{\alpha /2}^{2}\times {\mathrm{Se}}_{e}\times \left(1-{\mathrm{Se}}_{e}\right)}{d^{2}\times \mathrm{Pr}\mathrm{ev}},\frac{z_{\alpha /2}^{2}\times {\mathrm{Sp}}_{e}\times \left(1-{\mathrm{Sp}}_{e}\right)}{d^{2}\times \left(1-\mathrm{Pr}\mathrm{ev}\right)}\right)$$

where α is the significance level, Prev is the prevalence of the disease in the population and *d* is the precision of estimate (i.e.,the maximum marginal error). The number of subjects in the case (n_case_) and control (n_controls_) categories could be then estimated using Eq.(2):

(2) $$n_{\mathrm{controls}}=N\times \left(1-\mathrm{Pr}\mathrm{ev}\right);\;n_{\mathrm{case}}=N-n_{\mathrm{controls}}$$

The parameters Se_e_ and Sp_e_ were set to 70% and 77%, respectively based on the literature [20]. The prevalence of dyslipidemia in Iranian population was hypothesized as about 42% [6,28] and parameters α and *d* were both set to 0.05 [29]. Thus, the sample size of 725 (n_controls_ = 418, n_case_ = 307), sufficed.

### 2.2 Procedure and measurements

#### 2.2.1 DNA extraction

Single nucleotide polymorphisms (SNPs) of lipoprotein lipase LPL (D9N [rs1801177]), cholesteryl ester transfer protein CETP (TaqIB [rs708272]) [30], LPL (HindIII [rs320]), LPL (S447X [rs328]) [31], ATP-binding cassette transporter-1 ABCA1 (V771M [rs2066718]), ABCA1 (R1587K [rs2230808]) [32], CETP (A373P [rs5880]) [33,34], apolipoprotein C-3 APOC3 (*Sst*I [rs5128]) [35], apolipoprotein A-1 APOA1 (MspI [rs2893157]) [36], apolipoprotein A-5 APOA5 (C-1131T [rs662799]) [37] and apolipoprotein-E ApoE genes [38,39], appearing to relate to lipid profile disorders and (or) cardiovascular diseases, were investigated [3,40].

Subjects' peripheral blood was analyzed using the QIAamp DNA Blood Mini kit (Qiagen, Germany) and DNA was extracted following the manufacturer's protocol [41]. Corbett rotor-gene 6000 instruments (Corbett Research Pty Ltd, Sydney Australia) were used for Real-time PCR and high- resolution melt analysis [42]. The details of later analysis were mentioned in the Supplementary material S1.

Alleles of the genotypes were analyzed. Typically, only two out of the four possible nucleotides occur, and each sample contains a pair of every autosome. Alternatively, the carrier and non-carrier genes were represented as a binary variable for each genotype. For example, for the SNP rs320, nucleotide pairs GG, and TG/GT with the minority nucleotide G were considered as 'carrier' while the TT pair was set to 'non-carrier'. Thus, two feature sets (nucleotide pairs, and carrier/non-carriervariables) were considered for further analysis.

#### 2.2.2 Other analyzed features

The Anthropometric information was recorded by a team of trained health care professionals and the examinations were conducted under standard protocol by using calibrated instruments. Weight was measured to the nearest 200g in barefoot and lightly dressed condition. BMI was calculated as weight (kg) divided by height squared (m^2^). The parameter weight circumference (WC) was measured using a non-elastic tape to the nearest 0.2 cm at the end of expiration at the midpoint between the top of iliac crest and the lowest rib in standing position [25].

The anthropometric and life-style attributes such as age, sex, hypertension (either high systolic blood pressure (SBP) (≥90th percentile for age, sex and height) or high diastolic blood pressure (DBP) (≥90th percentile for age, sex and height) [43]), abdominal obesity (defined as waist-to-height ratio (WHtR) equal or more than 0.5 [44]), BMI categories (underweight, normal, overweight and obese defined using WHO growth curves [45] ) and physical activity (low, moderate, and severe categories [46]), as well as the family history of diabetes, obesity, CVD, cancer, and birth weight (<2500 g (low), 2500 g–4000 g (medium), and >4000 g (high) categories) were also included.

### 2.3 The proposed diagnosis system

#### 2.3.1 Pre-processing

The dataset was split into the estimation, validation (overall known as the training set) and test sets (40%, 10%, and 50% respectively in a hold-out validation setting). The input variables were grouped based on their interval or categorical measurement scales [47]. The categorical group consisted of nominal (such as sex) and ordinal (such as birth order) variables. The interval features were then transferred using robust Z-score measure [48,49]. In this transformation, the median and MAD (median absolute deviation) of each feature was estimated, and the median was then reduced from each feature and then normalized by the MAD value. Such features were then normalized between zero and one for further processing.

For each categorical feature, the indicator variable was estimated. It takes the value 0 or 1 to indicate the absence or presence of each category. Logit transformation was performed on each indicator variable whose intercept and slope parameters were estimated using maximum Likelihood Estimating (MLE) on the training set [50]. Thus, each indicator variable was expressed as a continuous value between zero and one. Such processed features are entitled as "predictors" from now on. The number of predictors was N_p_.

#### 2.3.2 Optimized inductive learning

Group Method of Data Handling (GMDH), first proposed by Ivakhnenko [51,52], has been applied in many areas for data mining [53]. Inductive GMDH algorithms find interactions in data, select an optimal network structure and thus improve the performance of current algorithms [54]. Here we proposed an optimized GMDH method to predict dyslipidemia using mixed-type data.

Feature selection was performed by iteratively estimating their weights based on their capability to discriminate between neighboring patterns in the framework of the Expectation-Maximization algorithm using I-RELIEF algorithm [55]. Moreover, the parallel selective sampling (PSS) method was used to select data from the majority class as to reduce the problems in the imbalanced datasets [56].

Multilayered induction for the gradual increase of complexity was performed using different layers. Instead of the fixed regression polynomial, the nonlinear regression matrix (*X*) was formed between any pairs (*i*,*j*) of predictors at the first layer that has *N*_*n*_ nonlinear regression functions:

(3) $$X_{N_{n}\times N_{1}}=\left[\begin{array}{c} a_{1}\times \mathrm{ones}\left(1,N_{1}\right)\\ a_{2}\times {\left(1+x\left(i,:\right)\right)}^{a_{3}}\\ a_{4}\times x{\left(j,:\right)}^{a_{5}}\\ \begin{array}{c} a_{6}\times \sin\left(a_{7}\times x\left(i,:\right)+a_{8}\times x\left(j,:\right)\right)\\ a_{9}\times x\left(i,:\right)⊙{\left(1+x\left(j,:\right)\right)}^{a_{10}}\\ \begin{array}{c} a_{11}\times \log_{2}\left(1+{\left|1+x\left(i,:\right)\right|}^{a_{12}}\right)\\ a_{13}\times \log_{2}\left(1+{\left|1+x\left(j,:\right)\right|}^{a_{14}}\right)\\ \ldots \end{array} \end{array} \end{array}\right]$$

where ⊙ is the element-by-element multiplication, a_i_ is the regression coefficients and *N*_*1*_ is the number of samples in the training set. If we fix the regression coefficients, the Regularized Least Squares (RLS) solution to *X*^*T*^ × *W* ≈ *B* (B is a column vector with the class label of the analyzed samples) could be estimated as below:

(4) $$W_{N_{n}\times 1}={\left(X\times X^{T}+\lambda \times I_{N_{n}\times N_{n}}\right)}^{-1}\times X\times B$$

where *λ* is the regularization parameter (set to 0.1 in our study), *I* is the identity matrix, and *T* is the matrix transpose operator. It could be easily shown that the optimal solution is the global minimum point of the RLS optimization [57]. In principle, it is possible to tune polynomial regression coefficients using a stochastic optimization [58]. Instead, we tune the regression coefficients used in the matrix *X*, using Particle Swarm Optimization (PSO). PSO is a meta-heuristicspopulation-based method inspired by flocking birds [59]. The topology and the internal parameters of PSO were the same as Mohebian et al. [15] except that the maximum number of iterations was set to 10 and the PSO fitness function was defined differently. At each PSO iteration, the random regression coefficients are used to calculate the matrix X for a predictor pair. Then, the parameter W is estimated on the training set. To avoid over-fitting, the estimated weight *W* is used on the validation set to estimate the output of the analyzed pair in the validation set. The cut-off of 0.5 was then used to estimate the parameters of signal detection theory such as True Positive (TP), True Negative (TN), False Positive (FP) and False Negative (FN). Then, parameters Sensitivity ($=\frac{\mathrm{TP}}{\mathrm{TP}+\mathrm{FN}}$), Specificity ($=\frac{\mathrm{TN}}{\mathrm{TN}+\mathrm{FP}}$) and Precision ($=\frac{\mathrm{TP}}{\mathrm{TP}+\mathrm{FP}}$) are estimated, and their average is used as the fitness function. The PSO method usually converged at few iterations due to the internal RLS optimization.

The selection pressure of the network was set to 0.7, in our study. Thus, 70% of the best pairs were selected for each layer. The approximating function of each selected pair was used as new features at the next layer [54]. The number of layers was estimated based on the required number of interactions. In a case of N_i_ interval features and N_d_ indicator variables, it was hypothesized as 1 + *round*(log_2_(*N*_*i*_ + *N*_*d*_)). At the last layer, the best approximation function was used as the output of the classification system. the overall structure of the proposed prediction system was shown in the Supplementary material S2.

### 2.4 State-of-the-art

In our study, other classification methods namely as multilayer perceptron (MLP), MLR and decision tree (DT), as proposed in other studies [19,20], were used for comparison. Supported vector machines (SVM) was also used for comparison. MLP, a feed-forward artificial neural network (ANN) model mapping sets of inputs onto a set of outputs [60], with one hidden layer with ten neurons and the sigmoid activation function [61] was used. SVM, constructing a hyper plane in a high-dimensional space [62], with the radial basis function (RBF) kernels were used. The soft-margin parameter and the radius of the RBF kernel were tuned using the method proposed by Wu and Wang [63]. DT, building classification models in the form of a tree structure [64], uses entropy to calculate the homogeneity of samples to build the tree. The statistical classifier C4.5 with pruning (i.e., removing redundant sub-trees) was used in our study [65]. The best splitting attribute is determined at each node. MLR uses the linear regression model with the Logit link function for the prediction.

After fitting the model [66], by estimating the model parameters, each case with the estimated class probability higher than 50%was classified as having dyslipidemia, or normal otherwise. In fact, DT and MLR could select relevant features because of the internal statistical validation. For MLP and SVM, Sequential Forward Selection (SFS) method, a bottom-up search procedure [67], was used for feature selection.

### 2.5 Validation

#### 2.5.1 The performance indices for each classifier

The performance of the classifiers was determined using the holdout method, where the dataset was split into two mutually exclusive sets (50% training and 50% test). The classifiers were then trained on the training set and tested on the test set [68]. Moreover, 4-foldcross-validation (60% estimation, 15% validation, 25% test in each analysis fold) was used to test the best classifiers to control a possible biased error estimate [67]. A variety of performance indices [15,69,70] were reported for the analyzed classifiers. Such indices along with their definitions were shown in Table1, among which, MCC is a single unbiased performance measure in balanced as well as imbalanced datasets [71]. It is related to chi-square statistics, also known as phi-coefficient, a measure of association for two binary variables (predicted versus observed gold-standard class) that could be interpreted as the correlation coefficient between those binary variables [72]. The interpretation of the reference intervals of the indices AUC ROC [73], Kappa [74], MCC [75] and DP [69,76] was listed in Supplementary material S3.

**Table1:** The classification performance measures used in our study.

$\mathrm{Se}=\mathrm{Rl}=\frac{\mathrm{TP}}{\mathrm{TP}+\mathrm{FN}}$	$\mathrm{Sp}=\frac{\mathrm{TN}}{\mathrm{TN}+\mathrm{FP}}$	$\mathrm{Acc}=\frac{\mathrm{TP}+\mathrm{TN}}{\mathrm{TP}+\mathrm{TN}+\mathrm{FP}+\mathrm{FN}}$
$\mathrm{Pr}=\frac{\mathrm{TP}}{\mathrm{TP}+\mathrm{FP}}$	*FA* = *α* = 1 − *Sp*	*Power* = 1 − *β* = *Se*
$F_{1}S=\frac{2\times \mathrm{Pr}\times \mathrm{Rl}}{\mathrm{Pr}+\mathrm{Rl}}$	$\mathrm{AUC}=\frac{\mathrm{Se}+\mathrm{Sp}}{2}$	${\mathrm{LR}}^{+}=\frac{\mathrm{Se}}{1-\mathrm{Sp}}$
${\mathrm{LR}}^{-}=\frac{1-\mathrm{Se}}{\mathrm{Sp}}$	$\mathrm{DOR}=\frac{{\mathrm{LR}}^{+}}{{\mathrm{LR}}^{-}}$	$\mathrm{DP}=\left(\sqrt{\frac{3}{\pi }}\right)\times \log\left(\mathrm{DOR}\right)$
$\mathrm{MCC}=\frac{\mathrm{TP}\times \mathrm{TN}-\mathrm{FP}\times \mathrm{FN}}{\sqrt{\left(\mathrm{TP}+\mathrm{FP}\right)\times \left(\mathrm{TP}+\mathrm{FN}\right)\times \left(\mathrm{TN}+\mathrm{FP}\right)\times \left(\mathrm{TN}+\mathrm{FN}\right)}}$	Kappa = agreement rate	

True positive (TP): subjects with dyslipidemia, correctly identified; false positive (FP): subjects without dyslipidemia, incorrectly identified; true negative (TN): subjects without dyslipidemia, correctly identified; false negative (FN): subjects with dyslipidemia, incorrectly identified; Se: sensitivity; Rl: recall; Sp: specificity; FA: false alarm; Acc: accuracy; Pr: precision; F_1_S: F1-Score; AUC: area under the receiver operating characteristic (ROC) curve; LR: likelihood ratio; DOR: diagnosis odds ratio; MCC: Matthews correlation coefficient; DP: discriminant power; Kappa: Cohen's kappa coefficient defined as the agreement rate between the predicted class labels and the gold standard.

A diagnosis system was considered as clinically reliable based on its Type I and II statistical errors [77], False Discovery Rate (FDR = 1-Precision) [78], and DOR [79] as to fulfill --all-- the following conditions: the minimum Sensitivity, Specificity, Precision and DOR of 80%, 95%, 95% and 100, respectively.

#### 2.5.2 Comparison between different classifiers

When different classifiers are compared with the gold standard, the superiority of one method to another must be presented using a proper statistical test. Otherwise, insignificant improvements might be erroneously reported as important [70]. McNemar's test, also known as the Gillick test, was used to compare the performance of two classifiers [67,80].

### 2.6 Statistical analysis

Results are reported as mean ± standard deviation (for interval variables) and frequencies (for categorical variables).The pairwise *χ*^2^ analysis was used to test for allele frequency differences (and nominal features) between dyslipidemia and normal groups and when the Cochran conditions were not met, the Fisher exact test was used. The *χ*^2^ analysis was used to test genotype frequency deviations from what predicted by the Hardy Weinberg equation. P-values less than 0.05 were considered significant. The entire data processing was performed off-line using Matlab version 8.6 (The MathWorks Inc., Natick, MA, USA). The statistical analysis and calculations were performed using the SPSS statistical package, version 16.0 (SPSS Inc., Chicago, IL, USA).

## 3 Results

The average age of the participants was 14.66 ± 2.61 years. Among the number of 725 patients participated in our study, 42.34% had dyslipidemia. Characteristics of the participants, grouped by their classification with/without dyslipidemia, are depicted in Table2. SNP genotype and allele frequencies in the study population were shown in Table3. None of the SNP distributions showed the deviation from Hardy-Weinberg equilibrium. Moreover, nucleotide pairs (Table3) showed better discrimination compared with carrier/non-carrier variables. Thus, nucleotide pairs, were used for prediction.

**Table2:** Characteristics of the participants in the dyslipidemia and normal groups.

		Dyslipidemia^⁎^			
Predictors	Categories	No	Yes	OR [CI 95%]	P-value
Age (years)		14.28 ± 2.26	14.64 ± 2.39	–	0.058
Sex	Male	49.28	46.61	0.90 [0.67,1.21]	0.477
	Female			–	
Region	Urban	64.80	71.71	1.38 [1.01,1.89]	0.049
	Rural			–	
Family history of diabetes	No	70.54	66.14	–	0.207
	Yes			1.23 [0.89,1.68]	
Family history of obesity	No	68.32	70.12	–	0.604
	Yes			0.92 [0.67,1.27]	
Family history of cancer	No	83.23	78.88	–	0.137
	Yes			1.33[0.91,1.93]	
Family history of CVD	No	87.16	92.43	–	0.023
	Yes			0.55 [0.33,0.93]	
Abdominal obesity	No	88.41	61.59	–	<0.001
	Yes			4.76 [3.26,6.94]	
BMI category (WHO criteria)	Under weight	25.85	19.52	0.76 [0.52,1.09]	0.007
	Normal	58.22	58.17	-	
	Over weight	8.36	10.76	1.29 [0.77,2.15]	
	Obese	7.57	11.55	1.53 [0.91,2.56]	
Physical activity	Mild	25.47	45.82	2.03 [1.43,2.87]	<0.001
	Moderate	40.37	35.86	–	
	High	34.16	18.32	0.60 [0.40,0.89]	
Birth weight	Low	11.67	16.73	1.54 [1.0,2.34]	0.249
	Normal	79.58	74.10	–	
	High	8.75	9.17	1.13 [0.67,1.89]	
Systolic blood pressure (mm Hg)		101.87 ± 13.16	104.16 ± 13.09	–	0.025
Diastolic blood pressure (mm Hg)		65.89 ± 10.74	66.69 ± 10.61	–	0.338
Fast blood sugar (mg/dL)		87.6 ± 11.85	84.32 ± 11.85	–	0.002
HDL-C (mg/dL)		59.95 ± 18.22	29.40 ± 12.37	–	<0.001
LDL-C (mg/dL)		75.43 ± 28.35	92.55 ± 38.09	–	<0.001
Total cholesterol (mg/dL)		149.66 ± 29.50	154.46 ± 30.20	–	0.061
Triglyceride (mg/dL)		86.06 ± 33.08	93.35 ± 34.35	–	<0.001

*: Results are reported as mean ± standard deviation (for interval variables) and percentage (for categorical variables). CVD: cardio-vascular disease; BMI: body mass index; WHO: world health organization; HDL-C: high-density lipoprotein cholesterol; LDL-C: low-density lipoprotein cholesterol; OR: Odds ratio (a categorical level was set to reference for each categorical variable); CI: confidence interval. In each dyslipidemia group, the frequency percentage of one of the categories in binary variables was shown.

**Table3:** SNP genotype and allele frequencies (in percentage) of the participants in the dyslipidemia and normal groups.

Polymorphism	Genotype and allele^⁎^	Dyslipidemia		OR [CI 95%]	P-value
		No	Yes		
LPL D9N [rs1801177]	AA	96.4	91.2	–	0.003
	AG			2.59 [1.35–4.96]	
ABCAI V771M [rs2066718]	GG	94.0	98.7	-	0.002
	GA			0.21 [0.07–0.60]	
LPL HindIII [rs320]	GG	24.4	50.8	–	<0.001
	GT	48.6	42.0	0.31 [0.23–0.43]	
	TT	27.0	7.2		
LPL S447X [rs328]	CC	72.7	88.6	–	<0.001
	CG	24.6	10.4	0.34 [0.23–0.52]	
	GG	2.6	1.0		
ABCAI R1587K [rs2230808]	AA	66.7	47.6	–	<0.001
	AG	29.9	39.4	2.21 [1.64–3.00]	
	GG	3.3	13.0		
CETP TaqIB [rs708272]	CC	19.1	60.6	–	<0.001
	CT	61.7	35.5	0.15 [0.11–0.22]	
	TT	19.1	3.9		
APOC3 *Sst*I [rs5128]	CC	83.0	83.7	–	0.371
	CG	16.7	15.3	0.95 [0.64–1.41]	
	GG	0.2	1.0		
CETP A373P [rs5880]	CC	93.5	77.9	–	<0.001
	CG	6.5	20.8	4.12 [2.56–6.62]	
	GG	0.0	1.3		
APOA1 MspI [rs2893157]	GG	69.4	74.3	–	0.119
	GA	27.8	24.8	0.79 [0.56–1.09]	
	AA	2.9	1.0		
APOA5 C-1131T [rs662799]	CC	98.8	97.7	–	0.525
	CT	0.5	1.0	1.93 [0.61–6.13]	
	TT	0.7	1.3		
ApoE	e2	6.9	0.7	1.73 [1.08–2.76]	<0.001
	e4	1.7	13.4		
	e3	91.4	86.0	–	

*: The alleles GG (SNP rs1801177) and CC (SNP rs2066718) had zero frequency in both normal and dyslipidemia groups and thus not shown in the results. OR: Odds ratio (a categorical level was set to reference for each categorical variable); CI: confidence interval. In each dyslipidemia group, the frequency percentage of one of the categories in binary variables was shown.

Three feature subsets were considered for prediction. Set 1 included sex, analyzed SNPs and family history of diseases: sex, LPL D9N [rs1801177], ABCAI V771M [rs2066718], LPL LPL HindIII [rs320], LPL S447X [rs328], ABCAI R1587K [rs2230808], CETP TaqIB [rs708272], APOC3 SstI [rs5128], CETP A373P [rs5880], APOA1 MspI [rs2893157], APOA5 C-1131T [rs662799], ApoE, Family history of diabetes, obesity, cancer, and CVD. Set 2 included Set 1 and birth weight, age, and physical activity. We also considered set 3 in which easily-measured features were analyzed, i.e., sex, age, physical activity, birth weight, BMI category, abdominal obesity, family history of diabetes, obesity, cancer, and CVD. The hold-out (50%) validation of the proposed method as well as the base learners DT, MLP, MLR, and SVM were performed in each feature subset, and the results of the classifiers on the test set were shown in Table4.

**Table4:** The hold-out (50%) validation of the classifiers.

Feature subset	Classifier	Se %	Sp %	Acc %	F_1_S %	Pr %	FA	AUC	MCC	DOR	DP	Kappa
1	Proposed	85	91	88	86	87	0.09	0.88	0.76	57	1.0	0.76
	DT	69	80	75	70	72	0.20	0.75	0.47	9	0.5	0.46
	MLP	67	88	79	73	80	0.12	0.78	0.56	15	0.6	0.56
	MLR	61	86	75	68	76	0.14	0.74	0.49	10	0.5	0.49
	SVM	71	78	75	70	70	0.22	0.75	0.45	9	0.5	0.44
2	Proposed	93	95	94	93	93	0.05	0.94	0.87	252	1.3	0.87
	DT	71	81	77	72	73	0.19	0.76	0.50	10	0.6	0.50
	MLP	70	86	79	74	79	0.14	0.78	0.57	14	0.6	0.57
	MLR	59	87	75	67	77	0.13	0.73	0.48	10	0.5	0.47
	SVM	71	82	77	72	74	0.18	0.77	0.52	11	0.6	0.52
3	Proposed	82	84	83	80	79	0.16	0.83	0.64	24	0.8	0.64
	DT	48	68	60	50	52	0.32	0.58⁎	0.12	2	0.2	0.10⁎
	MLP	17	93	61	27	64	0.07	0.55⁎	0.16	3	0.2	0.13⁎
	MLR	17	94	61	27	68	0.06	0.56⁎	0.18	3	0.3	0.14⁎
	SVM	61	68	65	59	58	0.32	0.65⁎	0.17	3	0.3	0.12⁎

Set 1 included sex, analyzed SNPs and family history of diseases: sex, LPL D9N [rs1801177], ABCAI V771M [rs2066718], LPL HindIII [rs320], LPL S447X [rs328], ABCAI R1587K [rs2230808], CETP TaqIB [rs708272], APOC3 SstI [rs5128], CETP A373P [rs5880], APOA1 MspI [rs2893157], APOA5 C-1131T [rs662799], ApoE, Family history of diabetes, obesity, cancer, and CVD. Set 2 included Set 1 and birth weight, age, and physical activity. Set 3 included sex, age, physical activity, birth weight, BMI category, abdominal obesity, family history of diabetes, obesity, cancer, and CVD. The classifiers were trained on the same training set and then validated on the test set and the results of the classifiers on the test set were shown.

⁎ Non-significant (P-value > 0.05).

In each feature subset, the proposed method significantly outperformed the base learners (DT, MLP, MLR, and SVM) (P-value < 0.05). In the third subset, the entire base learners did not reject the NULL hypothesis of an accidental agreement. Moreover, in such classifiers, the AUC ROC was not significant (P-value < 0.05) showing that none of them performed properly on the third subset. The proposed classifier on the set 2 significantly outperformed than sets 1 and 3 (P-value < 0.05). Also, the results of Set 1 was significantly better than those of Set 3 (P-value < 0.05).

The selected features of the proposed classifier on the Set 1 were CETP TaqIB [rs708272], CETP A373P [rs5880], LPL D9N [rs1801177], ApoE, ABCAI R1587K [rs2230808], APOA5 C-1131T [rs662799], LPL HindIII [rs320], APOC3 SstI [rs5128], family history of obesity, and diabetes, and APOA1 MspI [rs2893157]. Such features for Set 2 were CETP TaqIB [rs708272], ApoE, LPL D9N [rs1801177], ABCAI R1587K [rs2230808], age, birth weight, family history of obesity and for Set 3 were abdominal obesity, birth weight, physical activity, family history of diabetes, and BMI category. The performance of the best classifiers in each subset (i.e. the proposed classifier) was further assessed using 4-fold cross validation (Table5).

**Table5:** The four-fold cross validation results of the proposed prediction system in MEAN ± SD.

Feature subset	Se %	Sp %	Acc %	Pr %
1	87± 2	90 ± 1	89 ± 1	86 ± 1
2	93± 2	94 ± 1	94 ± 1	92 ± 1
3	83± 2	84 ± 2	84 ± 1	79 ± 2

Se: sensitivity; Sp: specificity; Acc: accuracy; Pr: precision.

The proposed prediction system showed limited discriminant power (DP = 1.3), excellent diagnosis accuracy (AUC ROC = 0.94), excellent agreement with the gold standard (Kappa = 0.87) and high correlation with the gold standard (MCC=0.87) on the second subset (Table4). The average statistical power and Type I error (α) were 93 % and 0.07, respectively based on the cross-validation on the second subset (Table5). The training time of the proposed system was 26.1 ± 2.2 (s), 33.6 ± 3.0 (s) and 20.5 ± 3.1 (s)in the first, second, and third subsets, respectively. The average running time was the average of 3 runs over 363 subjects in the training set (hold-out 50%) on an Intel Core i7-6500uCPU with 8 GB of RAM.

## 4 Discussion

Identifying high-risk children based on gene polymorphisms (sets 1, and 2), at the first place, is useful for further dietary, and life-styletreatments and screening. Using life-style, anthropometric indicators and family history of diseases (set 3), on the other hand, could identify the high-risk population in low-income countries.

### 4.1 The risk factors of dyslipidemia

Although the environment is very important in the development of dyslipidemia, genetic components are also critical [81]. CETP TaqIB [rs708272] was selected by the proposed dyslipidemia prediction system in both sets 1 and 2. In the literature, Genome wide association studies (GWAS) in adults showed a high correlation between CETP and plasma lipid concentrations [82]. However, such an association is less distinct in children [33,83]. It was shown in the literature that such a mutation has the protective effect on dyslipidemia [33] and Myocardial Infarction (MI) [84]. This was in agreement with our findings, where the OR of CT/TT vs. CC was 0.15 (P-value<0.001) (Table3).

ApoE was also selected in both sets. ApoE, playing an important function in lipid metabolism, has three isoforms, Apo-e2, Apo-e3, and Apo-e4. They are in fact translated into three alleles of the gene. It was shown in the literature that ApoE , and particularly, its e4 isoform, is associated with plasma lipid parameters and CVD risks [85,86]. Similarly, in our study, the prevalence of dyslipidemia was 85% in subjects with ApoE-e4 isoforms. Moreover, the OR of e2/e4 vs. e3 was 1.73 (P-value < 0.001) (Table3).

ABCAI R1587K [rs2230808] was the other selected feature in both sets 1 and 2. Several ABCA1 gene polymorphisms including R1587K [rs2230808], were identified. Dean et al. showed that this SNP is associated with the HDL-C concentration [87], thus affecting dyslipidemia. In our study, the OR of AG/GG vs. AA was 2.21 (P-value < 0.001) (Table3). Thus, such polymorphisms increased the risk of dyslipidemia.

D9N [rs1801177] was the other commonly selected SNP in our study. Corsetti et al. showed that D9N is as a predictor of CVD risk directly and through its interaction with TaqIB [30]. In fact, LPL is involved with triglyceride-rich lipoprotein metabolism and lipoprotein remodeling including HDL [88,89]. Similarly in our study, the OR of (AG/GG vs. AA was 2.59 (P-value = 0.003) (Table3).

The family history of obesity was another common feature. Valdez et al. indicated that people who have one or more relatives with diabetes or CVD have a high risk of such problems [90]. Such diseases have common risk factors such as obesity and dyslipidemia sharing etiology [91]. FH of obesity, however, had poor agreement rate with FH of diabetes in our database (Cohen's Kappa = 0.24; P-value < 0.05). FH of diabetes was selected in the first and third subset, though. The prevalence of dyslipidemia in subjects without FH of obesity and diabetes were 43% and 41%, respectively.

Birth weight was a selected feature for the subsets 2 and 3. Rodríguez Vargas et al. showed that high birth weight is not a risk factor for hypercholesterolemia or HDL and LDL-cholesterol esters, but is positive for TG [92]. In our study the ORs of the low and high birth weight categories were more than one, but not significant (Table3). The prevalence of dyslipidemia in the abnormal and normal birth weight groups were 45% and 41%, respectively.

CETP A373P [rs5880] was selected in the first set. Agerholm-Larsen et al. indicated that such a polymorphism is associated with decreased HDL-C [93]. Heidari-Beni et al. showed that HDL-C levels were significantly lower among those with CETP A373P [rs5880] polymorphism [33]. In our study, the OR of CG/GG vs. CC was 4.12 (P-value < 0.001) (Table3).

APOA5 C-1131T [rs662799] was another selected SNP in the first set. Wang et al. indicated that this polymorphism is associated with dyslipidemia and the severity of CHD [94]. In our dataset, the OR of AG/GG vs. AA was 1.93, but it was not significant due to the small sample size of carrier genotypes (P-value = 0.525) (Table3).

Radha et al. found an association between LPL HindIII [rs320] SNP with low HDL-C and elevated TG levels [95]. Song et al. indicated a significant association between the APOC3 SstI [rs5128] polymorphism and higher levels of TG, TC, and LDL-C [35]. Albahrani et al. showed that APOA1 MspI [rs2893157] polymorphism is associated with CVD risk [36]. We did not find such an increased risk of dyslipidemia for LPL HindIII [rs320], APOC3 SstI [rs5128] and APOA1 MspI [rs2893157] SNPs. However, Odds (dyslipidemia| GG) was 1.5 in LPL HindIII [rs320] showing that this was possibly a good feature for the proposed classifier. Due to the small sample size of AA alleles in APOA1 MspI [rs2893157] and GG alleles in APOC3 SstI [rs5128] (Table3), no significant association between such polymorphisms and the risk of dyslipidemia was found.

Anthropometric indices such as BMI and WHtR were shown to be associated with dyslipidemia in children and adolescents in the literature [96]. In our study, people with abdominal obesity had 4.76 times risk of dyslipidemia (OR = 4.76; P-value < 0.001) compared with those without such an obesity (Table2). Moreover, overweight and obese subjects had a higher risk of dyslipidemia compared with normal BMI subjects (Table2). In fact, WHtR and BMI were moderately correlated (r = 0.737; P-value < 0.001). WHtR was poorly correlated with TG (r = 0.257; P-value < 0.001) while BMI was poorly correlated with SBP (r = 0.248; P-value < 0.001) and TG (r (Pearson's correlation) = 0.293; P-value < 0.001). They could be the reason why BMI and WHtR were selected by the proposed classifier on the third set.

Panagiotakos et al. showed that lipid profile disorders are correlated with physical activity [97]. In our dataset, the ORs of high and low physical activity compared with moderate activity were 0.60 (P-value < 0.001) and 2.03 (P-value < 0.001), respectively (Table2). It was poorly correlated with HDL levels (*ρ* (Spearman's correlation) = 0.252; P-value < 0.001). That could support its selection on the third set. Age was selected in the second set. Age was shown to be an independent predictor of dyslipidemia in children and adolescents [26]. Although age was directly used in the second set, age and sex are indirectly required for dyslipidemia prediction on the thirst set. The identification of BMI category in children and adolescents is dependent on the growth-curve charts that are gender and age specific [45].

### 4.2 Application in health policy making

The proposed automatic diagnosis of dyslipidemia on the third set is indeed an effective screening system. It used the input features of abdominal obesity, birth weight, physical activity, family history of diabetes, and BMI category. It includes therapeutic life-style change (e.g., dietary therapy, and increased physical activity), before necessary pharmacologic interventions [98]. In fact, the primary treatment for dyslipidemia in children and adolescents is such a life-style change [26].

Although the proposed system on the set 3 it is not a fully clinically reliable system (Type I error of 16% and FDR of 21%), it could be possibly used in low- and middle- income countries where genomics is not possible for a large population. Moreover, embedding the prediction system into a public online web-interface is useful in health promotion programs [15,99] that will be the focus in our future work.

### 4.3 The Properties and Performance of the proposed system

The proposed system for dyslipidemia prediction in the subset 2, showed promising results regarding variety of performance indices (Table4, Table5). The statistical power, Type I error, FDR and DOR of the proposed system were 93%, 0.05, 7%, 252 (Table4). Thus, the proposed system fulfilled the criteria of a clinically reliable system except that it surpassed the minimum required FDR of 5% by 2%. We considered a variety of performance indices introduced in the literature (Table1, Table2), and also the Standards for Reporting Diagnostic Accuracy (STARD 2015) and its extensions [70,100] in reporting the results. Guarding against testing hypotheses suggested by the data (Type III errors [101]) done by cross-validation and the low variation (high consistency) of the performance indices in different folds (Table5), excellent balanced diagnosis accuracy (AUC ROC = 0.94), excellent class labeling agreement rate (Kappa = 0.87), high correlation between predicted and observed class labels (MCC = 0.87), limited discriminant power (DP = 1.3) (Table2, Table4), it is promising for clinical diagnosis tests. It significantly outperformed the other systems namely as DT, MLP, MLR, and SVM (McNemar's test; P-value<0.05).

Selecting only one kind of lipid disorder such as high total cholesterol/HDL-C ratio rather than dyslipidemia, could facilitate the interpretation of the results [20]. However, dyslipidemia contributes to cardio-metabolic risks in children and adolescents [102]. Moreover, In addition to cholesterol and HDL-C [103], triglyceride [104] and LDL-C [105] were shown to be important CVD risk factors. Thus, the outcome of the proposed system was dyslipidemia. We also considered high total cholesterol/HDL-C ratio outcome in our study and the selected features in the feature set 1 were ABCA1 (R1587K [rs2230808]), CETP (A373P [rs5880]), LPL (HindIII [rs320]), LPL (D9N [rs1801177]), and CETP (TaqIB [rs708272]). The AUC of this model was 0.82 in the hold-out validation.

### 4.4 Further application of the proposed classification system

The proposed dyslipidemia prediction system made use of the following properties: I) mapping the mixed-data types to interval data using Logit function, II) RELIEF feature selection, III) PSS random sampling for imbalanced datasets, IV) the involvement of feature interactions proposed by GMDH, V) using the nonlinear regression matrix instead of a fixed regression polynomial, VI) using inner-loopRLS instead of LS, VII) using outer-loopPSO for stochastic optimization, VIII) using estimation, validation and test sets to avoid over-fitting, IX) internal cross validation on the training set (estimation plus validation set) to improve generalization capability, and X) proper cost function as the mean of Se, Sp, and Pr suitable for imbalanced data sets.

In fact, the proposed system could be regarded as a general framework for two-class classification of imbalanced mixed-type data given that it is successfully tested on different datasets. The following datasets were used for validation of the proposed framework: Wisconsin breast Cancer (BCW), Pima Indian Diabetes (PIM), Glass [106], and Hepatitis [107]. The performance of the proposed framework on such datasets was shown in Supplementary material S4.

### 4.5 Final considerations

The limitation of the current study is that it was a retrospective study. More sources of error are more common in such studies compared with prospective studies because of bias and possible confounders [108]. Also, the sample size must be increased as to improve the statistical power in our diagnosis system [109]. Moreover, instead of testing a small number of pre-specified genetic regions, performing GWAS could be used in the examination of a genome-wide set of genetic variants in the entire genome in different individuals. For instance, more-prevalent mutations in LDL receptor (LDLR) gene were associated with dyslipidemia such as familial hypercholesterolemia, which is associated with early severe atherosclerosis and CAD [110]. In our study, NHLBI guideline was used to define dyslipidemia in children and adolescents. However, other standards such as American Heart Association (AHA) guideline [111] exist. The AHA guideline has different cut-points for TG and HDL-C. It also does not have a non-HDL-C criterion. Using AHA guideline, the class labels might change; thus affecting the proposed classification system. Finally, external validation (i.e. assessing the performance of the model on datasets from different institutions) is required in addition to an internal validation (i.e. hold-out and cross-validation) [112]. Unlike Costanza and Paccaud who rightfully used external validation in assessing their proposed lipid-disorder prediction model [20], other studies such as Wang et al. [19] and our study in this field and many studies in the other data mining areas in the literature do have only traditional internal cross-validation. This is the other limitation of our study.

## 5 Conclusions

In conclusion, we proposed a computer-aided diagnosis system to predict dyslipidemia whose performance was assessed using different criteria and in different validation frameworks. It is accurate and precise and could be possibly used for screening and risk assessment in the health promotion programs for children and adolescents. The developed framework is available to interested readers upon request.

The following are the supplementary data related to this article.

Supplementary material S1 High-resolution melt analysis. Supplementary material S1

**Supplementary material S2:**
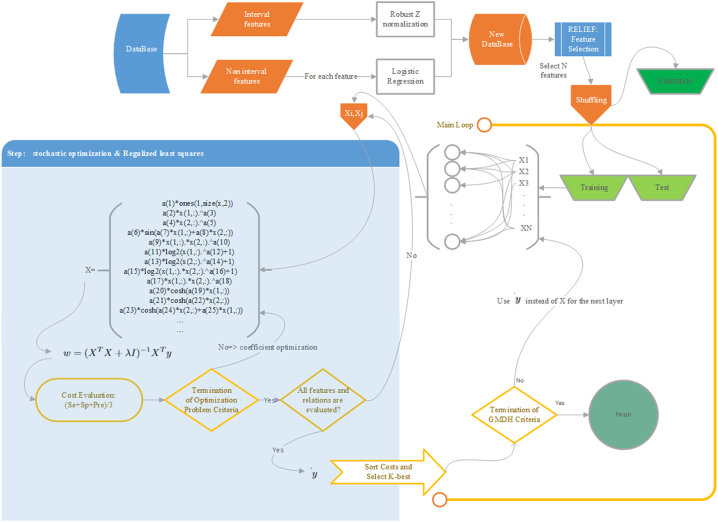
The flowchart of the proposed framework for classifying mixed-type data in imbalanced datasets

Supplementary material S3 The interpretation of the reference intervals of the performance indices used in this study. Supplementary material S3

Supplementary material S4 The performance assessment of the proposed framework on different datasets. Supplementary material S4
