# Supplementary material for: Prediction of dyslipidemia using gene mutations, family history of diseases and anthropometric indicators in children and adolescents: The CASPIAN-III study
Source: Comput Struct Biotechnol J. 2018 Mar 2;16:121–30. doi: 10.1016/j.csbj.2018.02.009 (PMC6050175; doi:10.1016/j.csbj.2018.02.009)
Supplement: Supplementary material S1 — High-resolution melt analysis. [file mmc1.docx]

# High-resolution melt analysis

Primers were designed by Beacon Designer 7.91 to flank the genomic regions (PREMIER Biosoft International, USA) and were synthesized by TIB MOLBIOL (Germany) [[1](#_ENREF_1), [2](#_ENREF_2)].

Amplicons were generated as the following conditions using type-it HRM kit (Qiagen, Germany): one cycle at 95°C (15 min); 40 cycles at 95 °C (15 sec), 60 °C (15 sec), 72 °C (15 sec); one cycle at 95 °C (15 min); 40 cycles at 95 °C (15 sec), 60 °C (15 sec), 72 °C (15 sec); one cycle of 95 °C (1 sec), 72 °C (90 sec) and a melt from 70 °C to 95 °C increasing at 0.1 °C per second [[1](#_ENREF_1), [3](#_ENREF_3)].

The amplification mixture of a total volume of 25 mL included 12.5 mL of HRM PCR master mix, 1.75 mL of ten mM primer mix, two mL of genomic DNA as the template and 8.25 mL of RNase-free water. For each genotype reaction, sequence-proven major and minor allele homozygote and heterozygote controls were included. The HRM analysis was performed by instrument software, allowing clustering of the samples into groups based on a difference plot obtained by analyzing the differences in melting curve shape between known controls and samples [[3](#_ENREF_3)].

# References

[1] Heidari-Beni M, Kelishadi R, Mansourian M, Askari G. Interaction of cholesterol ester transfer protein polymorphisms, body mass index, and birth weight with the risk of dyslipidemia in children and adolescents: the CASPIAN-III study. Iranian journal of basic medical sciences 2015;18: 1079-85

[2] Kelishadi R, Haghjooy Javanmard S, Tajadini MH, Mansourian M, Motlagh ME, Ardalan G, et al. Genetic association with low concentrations of high density lipoprotein-cholesterol in a pediatric population of the Middle East and North Africa: the CASPIAN-III study. Atherosclerosis 2014;237: 273-8.10.1016/j.atherosclerosis.2014.08.043.

[3] Askari G, Heidari-Beni M, Mansourian M, Esmaeil-Motlagh M, Kelishadi R. Interaction of lipoprotein lipase polymorphisms with body mass index and birth weight to modulate lipid profiles in children and adolescents: the CASPIAN-III Study. Sao Paulo Med J 2016;134: 121-9
