# Supplementary material for: Prediction of dyslipidemia using gene mutations, family history of diseases and anthropometric indicators in children and adolescents: The CASPIAN-III study
Source: Comput Struct Biotechnol J. 2018 Mar 2;16:121–30. doi: 10.1016/j.csbj.2018.02.009 (PMC6050175; doi:10.1016/j.csbj.2018.02.009)
Supplement: Supplementary material S3 — The interpretation of the reference intervals of the performance indices used in this study. [file mmc2.docx]

The interpretation of the reference intervals of the performance indices used in this study

| Performance Index | Property | The interpretation of the reference intervals |
| --- | --- | --- |
| AUC ROC | Balanced diagnosis accuracy | [0.7,0.8) good  [0.8,0.9) very good  [0.9,1.0] excellent |
| Kappa | Class labeling agreement rate | <0.40 poor  [0.40,0.75) fair to good  [0.75,1.00] excellent |
| MCC | Correlation between predicted and observed class labels | [0.0, 0.3) negligible  [0.3, 0.5) low  [0.5, 0.7) moderate  [0.7, 0.9) high  [0.9, 1.0] very high  (-1.0,0.0) negative (i.e. disagreement) |
| DP | Discriminant power | <1 poor  [1,2) limited  [2,3) fair $\geq$  3 good |

AUC ROC: area under the receiver operating characteristic curve; Kappa: Cohen's kappa coefficient; MCC: Matthews correlation coefficient; DP: discriminant power.
