# Supplementary material for: Prediction of dyslipidemia using gene mutations, family history of diseases and anthropometric indicators in children and adolescents: The CASPIAN-III study
Source: Comput Struct Biotechnol J. 2018 Mar 2;16:121–30. doi: 10.1016/j.csbj.2018.02.009 (PMC6050175; doi:10.1016/j.csbj.2018.02.009)
Supplement: Supplementary material S4 — The performance assessment of the proposed framework on different datasets. [file mmc3.docx]

**Table A1:** The performance assessment (hold out 70%) of the proposed framework on different datasets

| Set | Se  % | Sp  % | Acc % | Fscore  % | Pr  % | FA | Β | AUC | MCC | Prv  % | Number of attributes |
| --- | --- | --- | --- | --- | --- | --- | --- | --- | --- | --- | --- |
| BCW | 96 | 100 | 98 | 98 | 100 | 0.00 | 0.04 | 0.98 | 0.94 | 34 | 32 |
| PIM | 76 | 89 | 85 | 77 | 77 | 0.11 | 0.23 | 0.83 | 0.66 | 35 | 8 |
| Hepatitis | 73 | 85 | 80 | 77 | 80 | 0.15 | 0.27 | 0.79 | 0.60 | 45 | 19 |
| Glass | 88 | 94 | 92 | 85 | 83 | 0.06 | 0.12 | 0.91 | 0.81 | 24 | 10 |

Se: sensitivity; Sp: specificity; Acc: accuracy; Fscore: F_1_ score; Pr: precision; FA: false alarm (=α, Type I error); B= Type II error; AUC: the Area Under an ROC Curve; MCC: Matthews correlation coefficient; Prv: Prevalence of the minority class in the data set.

**Table A2:** The performance assessment (10-fold cross validation) of the proposed framework on different datasets

| **#F** | **Specificity**  **%** | **Sensitivity**  **%** | **Precision**  **%** | **Accuracy**  **%** | **Set** |
| --- | --- | --- | --- | --- | --- |
| 5 | 99.6±0.8 | 94.8±2.1 | 99.1±1.1 | 96.3±1.2 | BCW |
| 6 | 89.0±0.9 | 73.9±3.1 | 75.4±2.6 | 83.2±2.8 | PIM |
| 8 | 84.5±1.0 | 72.1±1.7 | 78.8±2.0 | 78.8±2.6 | Hepatitis |
| 6 | 93.2±0.9 | 87.1±1.2 | 81.5±2.1 | 90.8±1.8 | Glass |

#F: Number of selected features using the proposed framework.

**Datasets:**

**BCW** (Breast Cancer Wisconsin):

<https://archive.ics.uci.edu/ml/datasets/Breast+Cancer+Wisconsin+(Diagnostic)>

W.H. Wolberg, W.N. Street, and O.L. Mangasarian. Machine learning techniques to diagnose breast cancer from fine-needle aspirates. Cancer Letters 77 (1994) 163-171.

**PIM** (Pima Indians Diabetes):

<https://archive.ics.uci.edu/ml/datasets/pima+indians+diabetes>

Smith, J.W., Everhart, J.E., Dickson, W.C., Knowler, W.C., & Johannes, R.S. (1988). Using the ADAP learning algorithm to forecast the onset of diabetes mellitus. In Proceedings of the Symposium on Computer Applications and Medical Care} (pp. 261--265). IEEE Computer Society Press.

**Hepatitis**:

<https://archive.ics.uci.edu/ml/datasets/hepatitis>

Diaconis,P. & Efron,B. (1983). Computer-Intensive Methods in Statistics. Scientific American, Volume 248.

**Glass Identification**:

<https://archive.ics.uci.edu/ml/datasets/glass+identification>

Ian W. Evett and Ernest J. Spiehler. Rule Induction in Forensic Science. Central Research Establishment. Home Office Forensic Science Service. Aldermaston, Reading, Berkshire RG7 4PN.
